# Bacterial Cyclic AMP-Phosphodiesterase Activity Coordinates Biofilm Formation

**DOI:** 10.1371/journal.pone.0071267

**Published:** 2013-07-29

**Authors:** Eric J. Kalivoda, Kimberly M. Brothers, Nicholas A. Stella, Matthew J. Schmitt, Robert M. Q. Shanks

**Affiliations:** Charles T. Campbell Laboratory of Ophthalmic Microbiology, Department of Ophthalmology, University of Pittsburgh, Pittsburgh, Pennsylvania, Unites States of America; Ben-Gurion University of the Negev, Israel

## Abstract

Biofilm-related infections are a major contributor to human disease, and the capacity for surface attachment and biofilm formation are key attributes for the pathogenesis of microbes. *Serratia marcescens* type I fimbriae-dependent biofilms are coordinated by the adenylate cyclase, CyaA, and the cyclic 3′,5′-adenosine monophosphate (cAMP)-cAMP receptor protein (CRP) complex. This study uses *S. marcescens* as a model system to test the role of cAMP-phosphodiesterase activity in controlling biofilm formation. Herein we describe the characterization of a putative *S. marcescens* cAMP-phosphodiesterase gene (SMA3506), designated as *cpdS*, and demonstrated to be a functional cAMP-phosphodiesterase both *in vitro* and *in vivo*. Deletion of *cpdS* resulted in defective biofilm formation and reduced type I fimbriae production, whereas multicopy expression of *cpdS* conferred a type I fimbriae-dependent hyper-biofilm. Together, these results support a model in which bacterial cAMP-phosphodiesterase activity modulates biofilm formation.

## Introduction

The cyclic AMP (cAMP)-cAMP receptor protein (CRP) regulatory network exerts global control over processes of cellular physiology and virulence, such as cell division, catabolite repression, motility, and toxin production [1]–[3]. Bacterial catabolite repression modulates intracellular cAMP levels through adenylate cyclase (AC) and phosphoenolpyruvate: carbohydrate phosphotransferase (PTS) system activity [4], [5]. Degradation of cAMP to 5′-AMP is accomplished through enzymatic action of a cyclic 3′,5′-AMP phosphodiesterase (cAMP-PDE) [6]. The dynamic coordination of cAMP synthesis and degradation must be achieved for cAMP homeostasis and its effect on the cAMP-CRP network in response to fluctuating environments.

Many studies have highlighted an extraordinary role for ACs and cAMP-CRP in bacterial virulence-associated phenotypes. For instance, ACs and CRP-family proteins are required for full virulence in mouse infection models with the pathogens *Mycobacterium tuberculosis*, *Pseudomonas aeruginosa*, *Salmonella typhimurium*, and *Yersinia enterocolitica* [7]–[10]; in *Vibrio cholerae,* CRP is essential for *in vivo* colonization [11]. In *Serratia marcescens*, cAMP-CRP-dependent pathways control motility, hemolysis, production of flagella and the metalloprotease serralysin [12]–[14]. The function of cAMP-CRP is paramount to pathogenesis, yet the impact of altered cAMP equilibrium on surface attachment and biofilm formation is not well understood.

Biofilms, surface-adherent bacterial communities, are thought to afford microbes with the ability to persist in diverse environments, survive antimicrobials, and adapt to physiological stresses and host defenses [15]–[17]. Bacterial biofilms are of critical clinical significance and pose immense challenges to the eradication of multidrug-tolerant organisms, which likely support chronic and recurrent disease states that are recalcitrant to antimicrobial therapy [15], [18], [19]. An extensive spectrum of biofilm-related infections are implicated in human disease and foreign body-associated infections are intimately linked to biofilm formation [16]. Fimbriae, or type I pili, are crucial virulence factor(s) for biofilm development and bacterial attachment to host tissue surfaces [20]–[22]. Regulation of type I pili and/or biofilm formation by cAMP-CRP has been documented in *Escherichia coli* [23], [24] and *V. cholerae* [25], [26]. Moreover, *V. cholerae* PTS components are known to regulate biofilm formation [27].

*S. marcescens* engages several regulatory mechanisms for virulence, surface adhesion and biofilm formation [28]–[30]. Foremost among these are type I fimbrial adhesins [31], [32], which are critical mediators of surface attachment to biotic and abiotic surfaces [33], [34]. *S. marcescens* type I fimbriae-dependent biofilm formation is controlled by the cAMP-CRP complex; mutations inactivating positive regulators of cAMP, PTS enzyme IIA^Glc^ (*crr*) and adenylate cyclase (*cyaA*), or the master cAMP effector, CRP (*crp*), resulted in a hyper-biofilm phenotype [35]. Notably, the effect of negative cAMP regulators, specifically cAMP-PDEs, on biofilm formation has not been determined for any bacterial species. Using *S. marcescens* as a model system, we investigated the role for bacterial cAMP-PDE activity in biofilm formation.

## Materials and Methods

### Cultures and media

Bacteria were cultured at 30°C in Lysogeny broth (LB, per liter: 5 g yeast extract, 10 g tryptone, 5 g NaCl) in test tubes on a tissue culture rotor (New Brunswick model TC-7, speed setting 8, 62 rpm). Kanamycin was added to both agar and broth when appropriate at a concentration of 100 µg/ml, gentamicin at 10 µg/ml, and tetracycline at 10 µg/ml. *Saccharomyces cerevisiae* was grown in YPD or SC-URA [36]. Strains, plasmids and oligonucleotide primers are chronicled in Table 1, 2, and 3 respectively. The *S. marcescens* strain (CMS376) used in this study was obtained from the Presque Isle Culture Collection (PIC strain number 3611). This is a pigmented strain that has been used for previous biofilm studies [34], [35].

**Table 1:** Strains used in this study.

Strain	Description	Reference or source
InvSc1	*Saccharomyces cerevisiae* strain uracil auxotroph	Invitrogen
S17-1 λpir	*Escherichia coli* strain used for conjugation and cloning	[69]
EC100D	*E. coli* strain used for cloning and protein purification	Epicentre
ER2566	*E. coli* strain used for protein purification	New England Biolabs
JM3000-1	*E. coli* K-20 *rtlC cpdA::kan*	*E. coli* Genetic Stock Center
CMS376	*Serratia marcescens* wild type, PIC strain number 3611	Presque Isle Cultures
CMS524	*cyaA-2* – CMS376 with transposon mutation	[35]
CMS629	*fimC-2* – CMS376 with transposon mutation	[35]
CMS2910	CMS376 with *cpdS* deletion, Δ*cpdS*	This study
CMS3602	CMS2910 with *cyaA* transposon mutation (*prc-3*)	This study

**Table 2:** Plasmids used in this study.

Plasmid	Description	Reference or source
pMQ131	shuttle vector, *_ori_*pBBR1, *aphA-3*	[38]
pMQ132	shuttle vector, *_ori_*pBBR1, *aacC1*	[38]
pMQ158	pMQ132+ *cyaA*	this study
pMQ171	pMQ131+ *cpdS*	this study
pMQ176	pMQ131+ *cpdS-*N94A	this study
pMQ198	pMQ131+ *cpdS-*His_8_	this study
pMQ201	pMQ131+ *cpdS*, N94A-His_8_	this study
pMQ236	Allelic replacement vector, *_ori_*R6K, *nptII, I-SceI, rpsL*	[38]
pMQ240	I-SceI meganuclease expression vector for allelic replacement	[38]
pMQ247	pMQ131+ PA4969-His_8_	this study
pMQ360	pMQ236+ SMA3501-9 with *cpdS*-Δ deletion allele	this study
pEJK3	pMQ132+ *cpdA* from *E. coli*	[44]
pEJK5	pMQ132+ PA4969 from *P. aeruginosa*	this study
pEJK7	pMQ131+ *cpdA*-His_8_ from *E. coli*	this study

**Table 3:** Oligonucleotide primers used in this study.

Primer number	Primer sequence^a^
1140	caaattctgttttatcagaccgcttctgcgttctgatGCATCAGTATCCATCCGAGTCC
1141	tgtgagcggataacaatttcacacaggaaacagcttttATGGAAAGCCTGTTTAAACTGC
1359	GACCATCGCCGGTTGAAAATCGTGggcGCCCGGCAGCCACACGCAGG
1360	CCTGCGTGTGGCTGCCGGGCgccCACGATTTTCAACCGGCGATGGTC
1388	tctgttttatcagaccgcttctgcgttctgattcagtggtgatggtggtggtggtggtggTATCCATCCGAGTCCATATC
1615	aaattctgttttatcagaccgcttctgcgttctgatTCAGTATCCGGCGGTGTCGTAGTC
1616	attgtgagcggataacaatttcacacaggaaacagcttttATGTCACGCCATTCGAACAC
1619	aaattctgttttatcagaccgcttctgcgttctgatTCAGTAGCCTTCTGAAGCGGTATC
1620	gtgagcggataacaatttcacacaggaaacagcttttATGGAAAGCCTGTTAACCCTTCC
1687	ctgcgttctgattcagtggtgatggtggtggtggtggtgGTATCCGGCGGTGTCGTAGTC
1689	tgcgttctgattcagtggtgatggtggtggtggtggtgGTAGCCTTCTGAAGCGGTATC
2172	cagtgccaagcttgcatgcctgcaggtcgactctaGCTATGTTAGCGCTGACGATAGACG
2173	gtgcaggtagagcagcgtcgaaggcatcaATGGTGTCCTTTGTTATTTCAGTCTGTC
2175	gacagactgaaataacaaaggacaccatTGATGCCTTCGACGCTGCTCTACCTGCAC
2176	gctggagaaatgggaaagcatggcgcatCTGGAAAAACACCTGGCCATTGAGC
2178	gctcaatggccaggtgtttttccagATGCGCCATGCTTTCCCATTTCTCCAGC
2179	gataacaatttcacacaggaaacagctatgaccatgattCCGTTAACGCTAGCGCCAACG

a Lower case base pairs target recombination and upper case base pairs direct amplification.

### Biofilm and twitching assays

*S. marcescens* biofilms were formed on glass test tubes at high shear force as previously described [35]. Single colonies were added to 20 mm glass test tubes with 5 ml of LB and incubated with aeration on a TC-7 tissue culture rotor (New Brunswick Scientific) overnight at 30°C, a temperature that supports robust biofilm formation. Resulting biofilms adhering to the glass tubes were rinsed with tap water and stained using 5 ml of 0.1% crystal violet. The relative amount of biofilm was determined by solubilization of the crystal violet with 33% glacial acetic acid, and measurement of absorbance at 590 nm with a Synergy 2 plate reader (Biotek).

Viability of biofilms and planktonic cells was determined by staining cells with SYTO-9 and propidium iodide using a commercial kit and the manufacturer's specifications (BacLight L7012, Invitrogen). Static biofilms were formed on glass cover-slips that were incubated upright in a 12-well dish with 3 ml of LB inoculated with bacteria. The coverslip was dipped thrice in PBS to remove non-adherent cells and stained using the BacLight kit. Planktonic cells were from cultures grown overnight in LB with shaking, these were incubated and stained for viability and rinsed with PBS. A 10 µl aliquot was placed on the bottom of a glass-bottomed 6-well dish (Matek) followed by the addition of 37°C low melt agarose (25 µl of 0.5% agarose) to prevent movement of the cells during micrsocopy. Images were obtained using epifluorescent microscopy and experiments were performed on two different days with similar results.

### Molecular techniques

Plasmids were generated using yeast *in vivo* cloning [37]. To clone the SMA3506 (*cpdS*) open reading frame (ORF) from *S. marcescens*, primers (1140 and 1141) were designed to amplify SMA3506 with a change in the predicted start codon from “TTG” to “ATG”, for SMA3506 and all subsequent cAMP-PDE genes, to enhance translation efficiency. These primers also direct recombination into pMQ131 and pMQ132, which were linearized with SmaI. The recombination event places the SMA3506 ORF under control of the *E. coli* P*lac* promoter and the resulting plasmids were dubbed pMQ171 and pMQ172 respectively. The same strategy was used to clone *cpdA* (PA4969) from *P. aeruginosa* strain PA01 except that it was cloned into shuttle vectors pMQ132 using primers 1615 and 1616. Clones that conferred elevated PDE activity to crude lysates of *E. coli*, tested with bis(*p*-nitrophenyl) phosphate (bis-*p*NPP) as described below, were analyzed by PCR, sequenced and used for further analysis.

To make pMQ176 in which a N94A mutation was introduced into the SMA3506 ORF, the gene was amplified in two halves with overlapping regions into which the N94A mutation was engineered using primer sets 1141 with 1360, and 1140 with 1359. The two linear replicons were simultaneously recombined into pMQ131 using *in vivo* cloning. The His_8_-tagged versions of SMA3506 and SMA3506 *-N94A* were generated using 1388 and 1141. His_8_-tagged versions of the *cpdA* genes from *E. coli* and *P. aeruginosa* were generated with primer sets 1689 and 1620, and 1687 and 1616, respectively. Plasmids were sequenced to verify that unwanted mutations were not introduced during the PCR or recombination processes.

### Mutagenesis

In-frame deletion of the *cpdS* gene was accomplished by using the allelic replacement vector pMQ236 [38]. Briefly, primers 2172 and 2173 amplified a 1468 base pair amplicon upstream of *cpdS*, and primer sets 2175 and 2176 and 2178 and 2179 were used to amplify 3646 and 3468 base pair amplicons with an overlapping region of DNA (54 base pairs) downstream of *cpdS*. This large region was cloned in case the integration of pMQ236 during the first step of allelic replacement caused a polar effect on the likely essential gyrase genes downstream of *cpdS*. The pMQ236 was linearized by digestion with SmaI, and combined with the PCR amplicons in a *Saccharomyces cerevisiae* transformation reaction. Plasmids were isolated from the resulting Ura^+^ colonies and were introduced into *E. coli* strain S17-1 λpir. PCR was used to screen for plasmids with DNA upstream and downstream of *cpdS*, and the *cpdS* deletion was verified by sequencing. The resulting plasmid pMQ360 included 1468 base pairs of DNA upstream of *cpdS* and 7060 base pairs of DNA downstream of *cpdS*.

To delete the chromosomal copy of *cpdS*, pMQ360 was introduced into WT *S. marcescens* (CMS376) by conjugation and selection with kanamycin. Plasmid insertion at the *cpdS* locus was verified by PCR. The I-SceI expressing plasmid pMQ240 was introduced into the merodiploid strain to introduce a double strand break in the pMQ360 backbone. Kanamycin sensitive single colonies were isolated, and the *cpdS* gene status was assayed by PCR among the kanamycin susceptible candidates. Roughly half had an amplicon corresponding to the mutant allele and the rest to the expected size of the wild-type *cpdS* gene. The diagnostic amplicons were sequenced to verify the chromosomal *cpdS* deletion.

### Biochemical applications

Protein purification was performed using *E. coli* (S17-1 λpir) bearing plasmids with poly-histidine tagged versions of *cpdA*, PA4969, SMA3506, SMA3506-N94A, and the empty vector, pMQ131, for a mock-purification control. Cultures of bacteria (50–200 ml) were grown overnight in LB with kanamycin. Bacteria were pelleted by centrifugation and frozen at −80°C. Pellets were thawed on ice, disrupted in 15 ml lysis solution consisting of B-per lysis solution (Pierce), Halt-protease inhibitor, and DNase I using sonication. Lysates were clarified by centrifugation and aqueous fractions were passed over nickel-affinity columns using the manufacturers protocols (Pierce product no. 78100). PAGE analysis of eluted protein fractions indicate the presence of a predominant band that migrates at the expected size. Western blots using anti-histidine antibodies indicate the presence of a single band in the SMA3506-His_8_ and SMA3506-N94A-His_8_ samples that was absent in those made from the empty vector mock purification samples. Western blots were performed as previously described [39].

### Generation of a cpdS cyaA double mutant

Based on the prediction that a *cpdS cyaA* double mutant would behave as a *cyaA* mutant with respect to prodigiosin pigment production, we screened the *cpdS* strain that had been randomly mutagenized with a transposon for isolates with a precocious red colony phenotype. These were noted as precocious red colony mutants (PRC). A mariner-based transposon from plasmid pSC189 was introduced into *S. marcescens* strain CMS2910 (Δ*cpdS* mutant) as previously described to initiate mutagenesis. Ten independent conjugation pools were plated onto LB agar plates selective for *S. marcescens* that had experienced a transposon insertion, and these were incubated at 30°C. After 16–20 hours, plates were visually inspected for hyper-red colonies, and one was chosen from nine of the conjugation plates from a total of ∼4000 mutant colonies. Four of the nine PRC mutant isolates were chosen based upon their enhanced pigmentation, increased hemolysis on blood agar plates and increased biofilm production – phenotypes exhibited by *cyaA* mutants. A medium-copy plasmid bearing the wild-type *cyaA* gene (pMQ158) was introduced into the four candidates. Three of the four mutant strains were complemented for their pigment and biofilm phenotypes (hemolysis was not tested). When the *cyaA* plasmid was lost through serial passage without selective antibiotic, the *cyaA* mutant-like phenotypes were restored (data not shown). The transposon insertion site for the *cyaA*-plasmid complemented candidates was performed as previously described [40] using SacII to digest the genome and T4 DNA ligase. The circularized genome fragments containing the plasmid were selected with kanamycin in *E. coli* strain EC100D *pir-116* (Epicentre), plasmid minipreps were prepared and sequenced. Two of the mutants had transposons in the *cyaA* gene located at base pair 102 and 632 respectively; the other insertion site was not determined.

### *In vitro* cAMP phosphodiesterase assays

Phosphodiesterase activity conferred by cAMP-PDE genes was measured using the method of Kuchma, et al. [41] with minor alterations. *E. coli cpdA* mutant strain JM3000 was used. JM3000 with the empty vector or test plasmid was grown for 16–18 h in LB medium with selective antibiotic, 1 ml of culture was obtained and the bacteria were pelleted by centrifugation. Bacterial cells were washed with 1 ml of reaction buffer (5 mM MgCl_2_, 50 mM Tris-HCl pH 9.3, and 50 mM NaCl), suspended in 0.75 ml reaction buffer and sonicated on ice until the lysate cleared. Lysates were clarified by centrifugation (16,000×g for 15 m at 4°C) and the protein concentration of the supernatant was determined by Bradford analysis. Lysates were normalized to 100 µg/ml in 0.1 ml reaction buffer and mixed with 0.1 ml of 10 mM bis-*p*NPP (Sigma product number N3002). The release of *p*-nitrophenol was measured in 96 well plates using a plate reader (A = 410 nm). Experiments were performed with triplicate biological replicates on two different days.

A thin layer chromatography (TLC) based cAMP-PDE assay was used to separate cAMP from 5′-AMP. Briefly 15 µl of reaction mixture (10 µl of 30 mM cAMP in enzyme buffer (1 mM MnCl_2_, 1 mM FeCl_2_, 50 mM Tris-Cl, 50 mM NaCl, pH 8), 10 µl of protein or protein elution buffer as a negative control), and 5 µl of water were incubated for 1 hour at 37°C. Aliquots (5 µl) were spotted and dried on a silica gel HLF TLC plate (Analtech product no. 47021) and separated using an ethanol/water/ammonium bicarbonate mixture (70:30:0.2 M) as a solvent [42]. Separated nucleotides were visualized with short wave UV light and photographed with a digital camera. Heat inactivation of protein was performed by incubation of the reaction mixture at 95°C for 20 minutes. The experiment was performed at least three times with each protein on different days and with at least two independent preparations of each protein.

An *in vitro* cAMP-PDE assay, based on the method of Hosono [43], was developed in which cAMP was mixed with either a positive control cAMP-PDE or SMA3506 or SMA3506-N94A and with alkaline phosphatase (AP). The basis of the assay being that cAMP-PDE activity will generate 5′-AMP from cAMP which can then be cleaved by AP to generate phosphate, whereas AP does not generate free phosphate from cAMP (confirmed in pilot assays). Available phosphate can be measured with the ascorbic acid-molybdate method, which generates a colorometric output measurable at A_820_. A reaction mixture composed of 500 µl of solution A (50 mM Tric-Cl, 5 mM MnCl_2_, 5 mM FeCl_2_, 50 mM NaCl, pH8), 100 µl of solution B (20 mM MgSO_4_, 100 µl of 2 mM cAMP, 100 µl of alkaline phosphatase (Sigma P5931 –28 units/ml)), and 200 µl of test protein or mock control (mock purified protein from *E. coli* with empty vector). This reaction was incubated for 2 hours at 38°C, at which time 100 µl of 55% TCA was added to stop the reaction. An equal volume of coloring reagent (1.2 N H_2_SO_4_, 0.5% ammonium molybdate, and 0.2% ascorbic acid) was then used to measure phosphate concentration indicating cAMP-PDE activity. The mixture was incubated at 38°C for 90 minutes and absorbance was read at 820 nm. A standard curve of phosphate was generated and used to measure phosphate release by the purified proteins and control samples.

### Intracellular cAMP analysis

Cyclic-AMP concentrations were determined using an Enzyme Immunoassay (EIA) (Caymen Chemical-product number 581001) according to the manufacturers specifications using the acetylation protocol. *S. marcescens* strains were grown in LB to an OD_600_ of 4.3–5.0 and normalized to lowest culture OD_600_. Five ml of culture was collected and pelleted by centrifugation at 16,000×g for 1 minute. Bacterial pellets were washed three times in charcoal filtered phosphate-buffered saline (PBS) to remove extracellular cAMP. Pellets were lysed by sonication in 250 μl of charcoal filtered PBS (Fisher Scientific Sonic Dismembrator model number 100) on ice until lysates became visually clear.

Cell lysate protein concentrations were determined by Bradford Assay and normalized to the least concentrated sample (0.7–6.2 µg/ml of protein). 50 μl of lysate was used per well for EIA. cAMP concentrations (pmol/ml) were determined using a standard curve and online tools from Caymen chemical (www.myassays.com). Experiments were performed with at least three biological replicates per assay and repeated on at least two different days.

### Transmission Electron Microscopy (TEM)

Performed as previously described using overnight cultures of cells grown in LB broth, washed with PBS, spotted on formvar coated grids, strained with uranyl acetate (1%) and viewed with a JEM-1210 electron microscope. The experiment was performed at least two times per genotype with independent biological replicates. At least 50 images were taken at 10,000× magnification per genotype based on the presence of non-clumped bacteria. Cell length was determined using Image J Software.

### Yeast agglutination assay

These assays were performed as previously described [35], [44]. For the type I fimbriae-specific yeast agglutination assay bacteria were grown overnight in LB, washed in PBS and adjusted to OD_600_  = 1.0 in PBS. Yeast (Sigma product no. YSC2) was added to PBS at a concentration of 0.2 g/10 ml. To a 1 cm cuvette, PBS (1.5 ml), yeast (0.5 ml) and bacteria (0.4 ml) were added and shaken. Cuvettes were placed in a spectrophotometer and the OD_600_ was taken at time intervals up to 10 minutes. Experiments were done with triplicate to quadruplicate biological replicates and performed on at least two different days.

### Statistical Analysis

One-way ANOVA with Tukey's multiple comparison test was done for experiments with more than two groups. A two-tailed unpaired Student's T-test was performed for experiments with two groups. Fisher's exact test was used for experiments with categorical variables. Analysis was performed using Graphpad Prism 5.

## Results

### Multicopy expression of the *E. coli cpdA* gene stimulates *S. marcescens* biofilm formation

**Figure 1:**
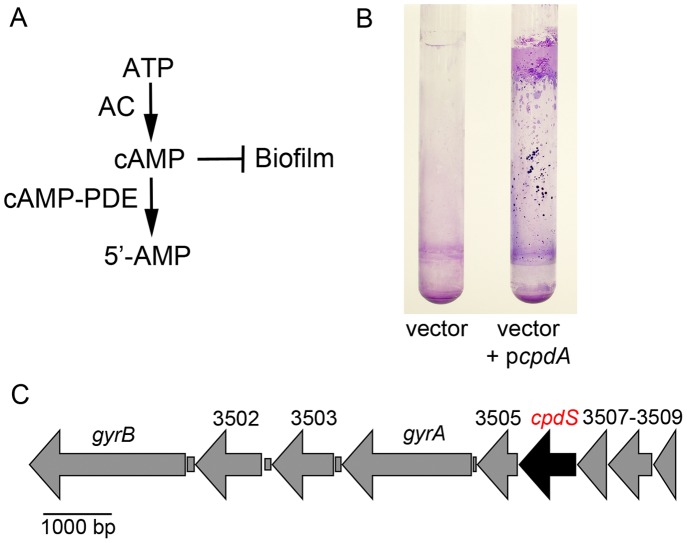
Role of cAMP-phosphodiesterase activity in biofilm formation and identification of a cAMP-phosphodiesterase gene in the *S. marcescens* genome. **A.** Model for cAMP metabolism and inhibitory effect on biofilm production. Adenylate cyclase (AC) catalyzes synthesis of cAMP from ATP, whereas cyclic-AMP phosphodiesterase (cAMP-PDE) catalyzes hydrolysis of cAMP to 5′-AMP. **B.** Crystal violet stained biofilms on the side of test tubes formed under high-sheer conditions. Shown is a wild-type *S. marcescens* strain with either the empty vector or the vector with a wild-type copy of the *E. coli* cAMP-PDE gene, *cpdA*. **C.** Genomic context of the *S. marcescens cpdS* gene, a candidate cAMP-PDE gene.

Based on previous studies, we developed a simple model for the role of cAMP as a regulator of biofilm formation by *S. marcescens* (Figure 1A). The primary goal of this study was to examine the contributions of cAMP-PDE activity on biofilm formation by *S. marcescens*. To test this, we cloned a known 3′,5′-cAMP-PDE gene (*cpdA*) from *E. coli* and placed it under control of the *P_lac_* promoter on a multicopy plasmid. The plasmid was introduced into *S. marcescens* and it conferred a dramatic increase in biofilm formation (Figure 1B). This result provided impetus to seek a native cAMP-PDE gene and to test the importance of this putative gene in biofilm formation.

### Identification of a candidate cAMP-PDE in *S. marcescens*

An open reading frame (ORF), SMA3506, was identified by blasting the *cpdA* gene from *E. coli* against a sequenced strain of *Serratia marcescens* (Sanger Center). The SMA3506 ORF will be referred to as *cpdS* for cyclic-AMP phosphodiesterase from *S. marcescens* as described below. This gene is the fourth open reading frame in a group of nine genes that are oriented in the same direction and may be in an operon (Figure 1C). This genomic context is similar to that of *cpdA* from *E. coli*, with inclusion of DNA gyrase, *gyrA*, in the putative operon [45]. The *cpdS* ORF was cloned from *S. marcescens* strain CMS376 to test if its protein product functions as a PDE and to determine whether this activity has a role in surface attachment. *cpdS* was placed under control of the *P_lac_* promoter on a pBBR1-based plasmid (pMQ171). *cpdS* from laboratory strain CMS376 was sequenced (GenBank number EU925585) and the DNA sequence was 96.3% identical to *cpdS* from the sequenced *S. marcescens strain*, Db11. Compared to five previously characterized class III 3′,5′-cAMP-PDEs, the CpdS predicted protein is 73% identical to the CpdA protein from *E. coli*, 70% identical to the CpdA protein from *K. pneumoniae*, 58% identical to the Icc protein from *Haemophilus influenza*, 42% identical to the Rv0805 gene from *Mycobacterium tuberculosis*, and 40% identical to CpdA from *Pseudomonas aeruginosa* (Figure S1). All of these proteins contain conserved residues for catalytic activity and metal binding and contain a conserved sequence for the active site from purple acid phosphatases (D-(X)n-GD-(X)n-GNH[E/D]-(X)n-H-(X)n-GHXH) that is necessary for PDE activity [46] (Figure S1).

### CpdS can hydrolyze cAMP *in vitro*

**Figure 2:**
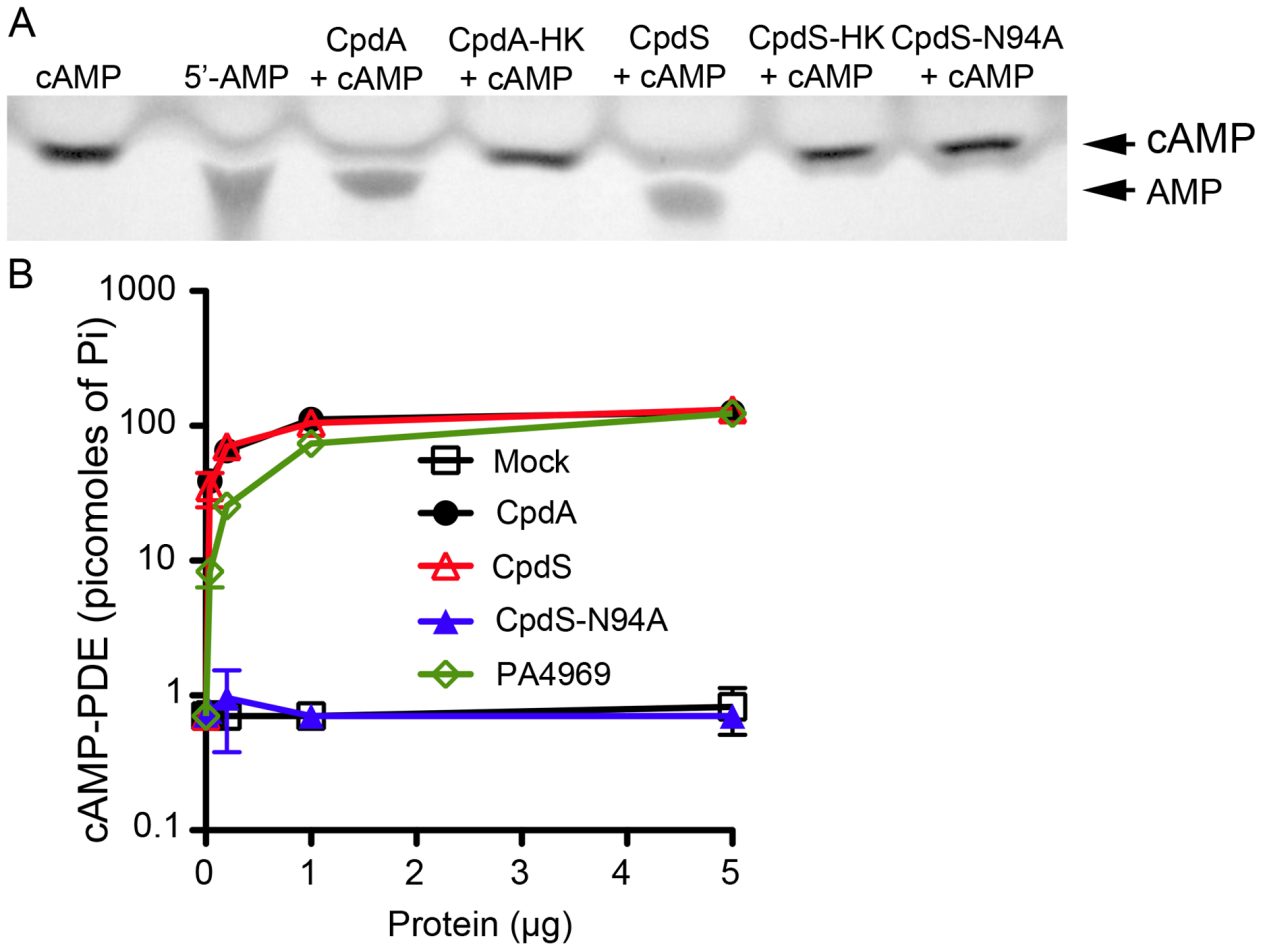
CpdS exhibits cAMP-PDE activity *in vitro*. **A.** Thin layer chromatography to examine cAMP-PDE activity from purified CpdA from *E. coli*, CpdS, and CpdS-N94A mixed with cAMP. HK  =  indicates heat killed protein before being mixed with cAMP. **B.** cAMP-PDE assay shows dose responsive activity by CpdS and positive controls CpdA and PA4969 (CpdA from *P. aeruginosa*), but not by the CpdS-N94A mutant or a mock purified protein.

To determine whether the protein encoded by *cpdS* could hydrolyze cAMP *in vitro*, we generated poly-histidine-tagged recombinant CpdS (Figure S2A). For a negative control, a mutant version (*cpdS*-N94A) with an alanine substitute for a highly conserved asparagine residue that is necessary for cAMP-PDE activity in CpdA of *P. aeruginosa* and *E. coli* was made (Figure S2A). Immunobots of crude lysates from *E. coli* and *S. marcescens* support that the recombinant CpdS and CpdS-N94A were stable (Figure S2B). CpdS-His_8_ and CpdS-N94A-His_8_ were incubated with cAMP and the resulting products were separated with thin layer chromatography (TLC). The production of 5′-AMP was observed when cAMP was mixed with the purified CpdA-His_8_ positive control protein from *E. coli* and CpdS-His_8_ (Figure 2A). Heat-killed cAMP-PDE protein, the no protein control, and the mutant CpdS-N94A-His_8_ protein were unable to hydrolyze cAMP (Figure 2A).

A second method was used to verify cAMP hydrolysis in which cAMP was placed in a reaction mixture with both test protein (CpdA, CpdS, or CpdS-N94A) and alkaline phosphatase (AP). In this reaction, if cAMP is hydrolyzed into 5′-AMP, AP can release inorganic phosphate from 5′-AMP, which can be measured colorometrically using the ascorbic acid-molybdate method [43]. The positive controls CpdA from *E. coli* and *P. aeruginosa* (PA4969) released phosphate, indicating cAMP-PDE activity (Figure 2B). CpdS-His_8_, unlike CpdS-N94A-His_8_ and the mock-purified protein control (Mock), exhibited dose-dependent cAMP-PDE activity (Figure 2B, Figure S2C).

We observed that the phosphodiesterase inhibitor IBMX inhibited CpdS-His_8_ in a dose dependent manner using the general phosphodiesterase substrate bis-*p*NPP to assess PDE activity [47], with 4.8 mM (4.1 to 5.6–95% confidence interval) of IBMX conferring a 50% reduction in the activity of 1 µg of CpdS-His_8_ (Figure S2D). These data indicate that the *cpdS* gene from *S. marcescens* codes for a phosphodiesterase able to hydrolyze cAMP *in vitro*.

### CpdS exhibits *in vivo* cAMP-PDE activity

**Figure 3:**
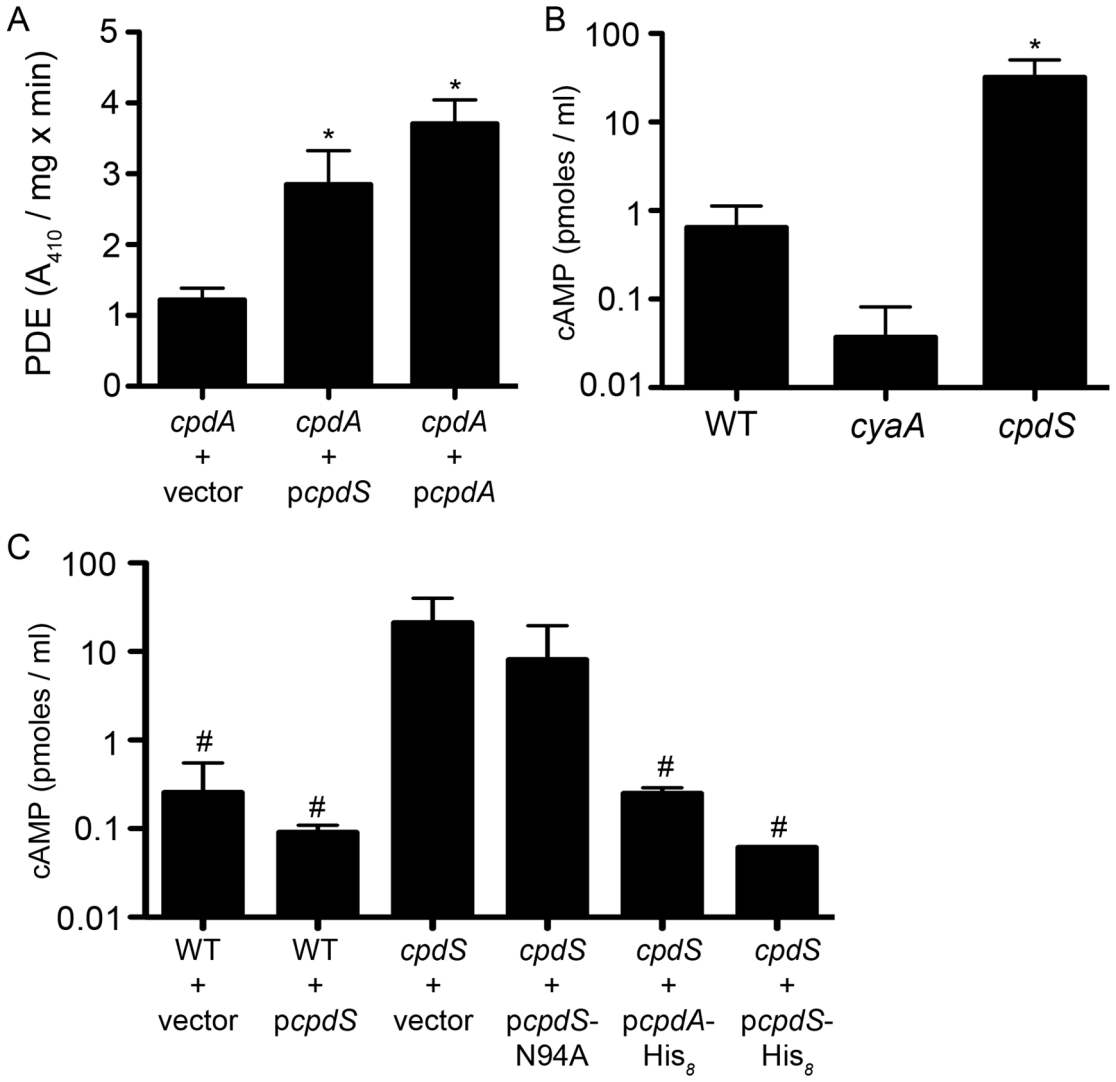
CpdS exhibits cAMP-PDE activity *in vivo*. **A.** Phosphodiesterase activity measured from protein lysates of an *E. coli cpdA* mutant strain bearing the negative control empty vector (pMQ131), p*cpdS* (pMQ171), or positive control p*cpdA* (pEJK3). Bis-*p*NPP was used as a substrate to measure PDE activity. **B–C.** Intracellular cAMP levels measured using an EIA assay was measured from *S. marcescens* cells of the indicated genotypes. Vector  =  pMQ131, p*cpdS*  =  pMQ171, p*cpdA*  =  pEJK3, p*cpdS*-N94A  =  pMQ176, p*cpdA*-His_8_  =  pEJK7, p*cpdS*-His_8_  =  pMQ198. Asterisk indicates significant increase compared to *cpdA* + vector (A) or WT (B), p<0.05 by ANOVA with Tukey's post-test. The number sign indicates a significant reduction from *cpdS* + vector, p<0.05 by ANOVA with Tukey's post-test.

We performed assays to test the hypothesis that CpdS codes for a functional cAMP-PDE *in vivo*. *E. coli* with a *cpdA* mutation bearing the vector alone, the vector with *cpdS*, and the vector with *cpdS-N94A* were tested for PDE activity. PDE activity was observed in lysates from cells with multicopy expression of *cpdS* (p*cpdS* plasmid) or the positive control *E. coli* plasmid (p*cpdA*), but not from lysates from the CpdS*-*N94A strains (p*cpdS-*N94A plasmid) (Figure 3A). Complementation of the cpdA mutation in E. coli with the *cpdS* gene demonstrated that multicopy expression of *cpdS* was sufficient to confer PDE activity *in vivo*.

To test whether CpdS has a role *in vivo* in *S. marcescens*, we mutated the *cpdS* gene by deletion of the *cpdS* open reading frame and measured intracellular levels of cAMP. The cAMP levels from the *cpdS* mutant were ∼50-fold higher in the *cpdS* deletion mutant strain than the WT, whereas cAMP levels from an adenylate cyclase mutant, CMS524, were near the minimum detection level of the assay and 17-fold lower than the WT (Figure 3 B–C). Episomal expression of *E. coli cpdA* or *S. marcescens cpdS* on a plasmid reversed the elevated cAMP levels of the *cpdS* mutant, whereas the *cpdS-*N94A mutant gene did not (Figure 3C). Together these results indicate that CpdS is a functional cAMP-PDE *in vivo*.

### CpdS mediates biofilm formation through type I fimbriae

**Figure 4:**
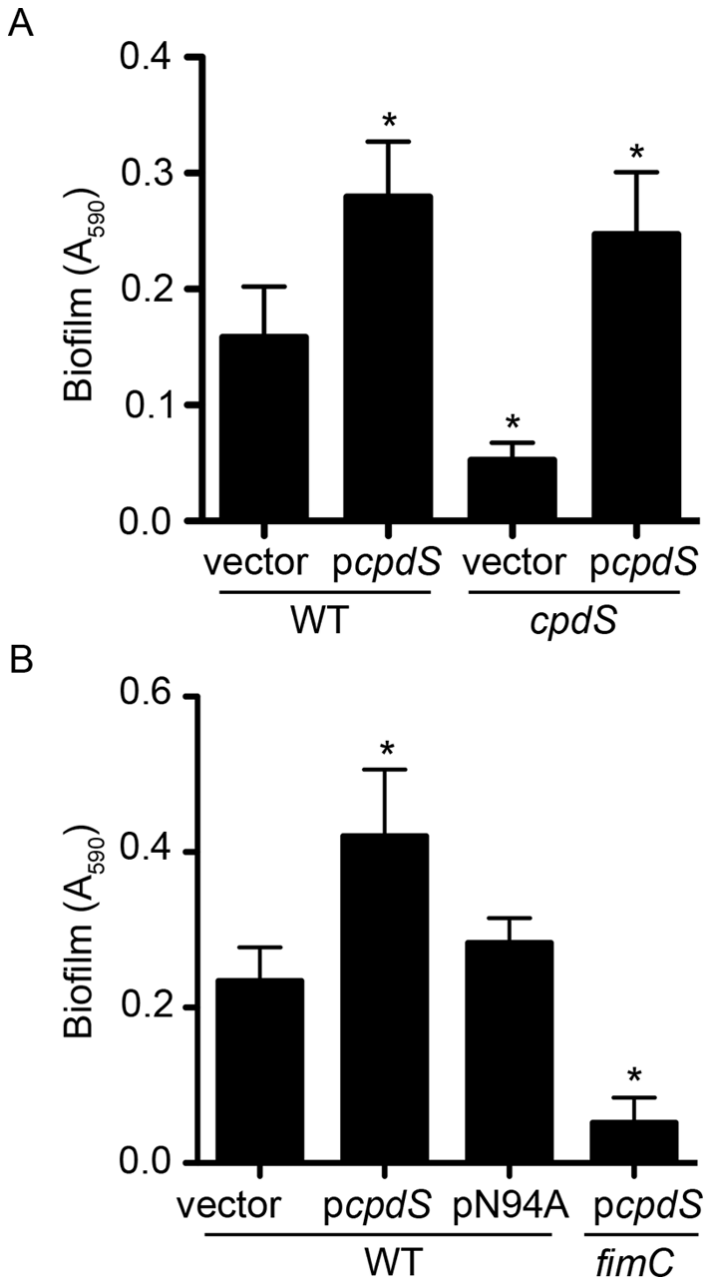
CpdS mediates *S. marcescens* biofilm formation. **A–B**. Quantification of crystal violet stained biofilms from the sides of glass test tubes grown under high-sheer conditions. Vector  =  pMQ131; p*cpdS*  =  pMQ171; pN94A  =  pMQ176. The asterisk indicates statistical difference from wild-type levels (n = 9, p<0.05, ANOVA with Tukey's post-test).

To determine whether physiological levels of CpdS mediate *S. marcescens* biofilm formation, we assessed biofilm formation in the *cpdS* deletion strain. A significant 66% reduction in biofilm formation was observed in the *cpdS* mutant compared to the WT (Figure 4A–B). As with *cpdA* (Figure 1B), multicopy expression of *cpdS* enhanced biofilm formation by both the WT and *cpdS* mutant strain (Figure 4A–B). Multicopy expression of the PDE defective cpdS-N94A mutant gene did not confer a hyper-biofilm phenotype (Figure 4B).

The viability of *cpdS* mutant and WT cells was assessed using fluorescence-based viability assay to test the prediction that altered cell viability contributed to the reduced biofilm exhibited by the *cpdS* mutant. No obvious difference was observed between planktonic or biofilm cells (Figure S3), indicating that another mechanism underlied the differential ability of these strains to form biofilms.

Because it was previously noted that *S. marcescens cyaA* and *crp* mutants develop hyper-biofilms and elevated production of type I fimbriae [35], we tested whether CpdS mediates biofilm formation through differential biosynthesis of fimbriae. Multicopy expression of *cpdS* in a *fimC* mutant, which is defective in the fimbrial usher component and unable to make surface fimbriae [35], did not confer a hyper-biofilm (Figure 4B). This shows that fimbriae are required for the hyper-biofilm phenotype conferred by multicopy *cpdS* expression.

**Figure 5:**
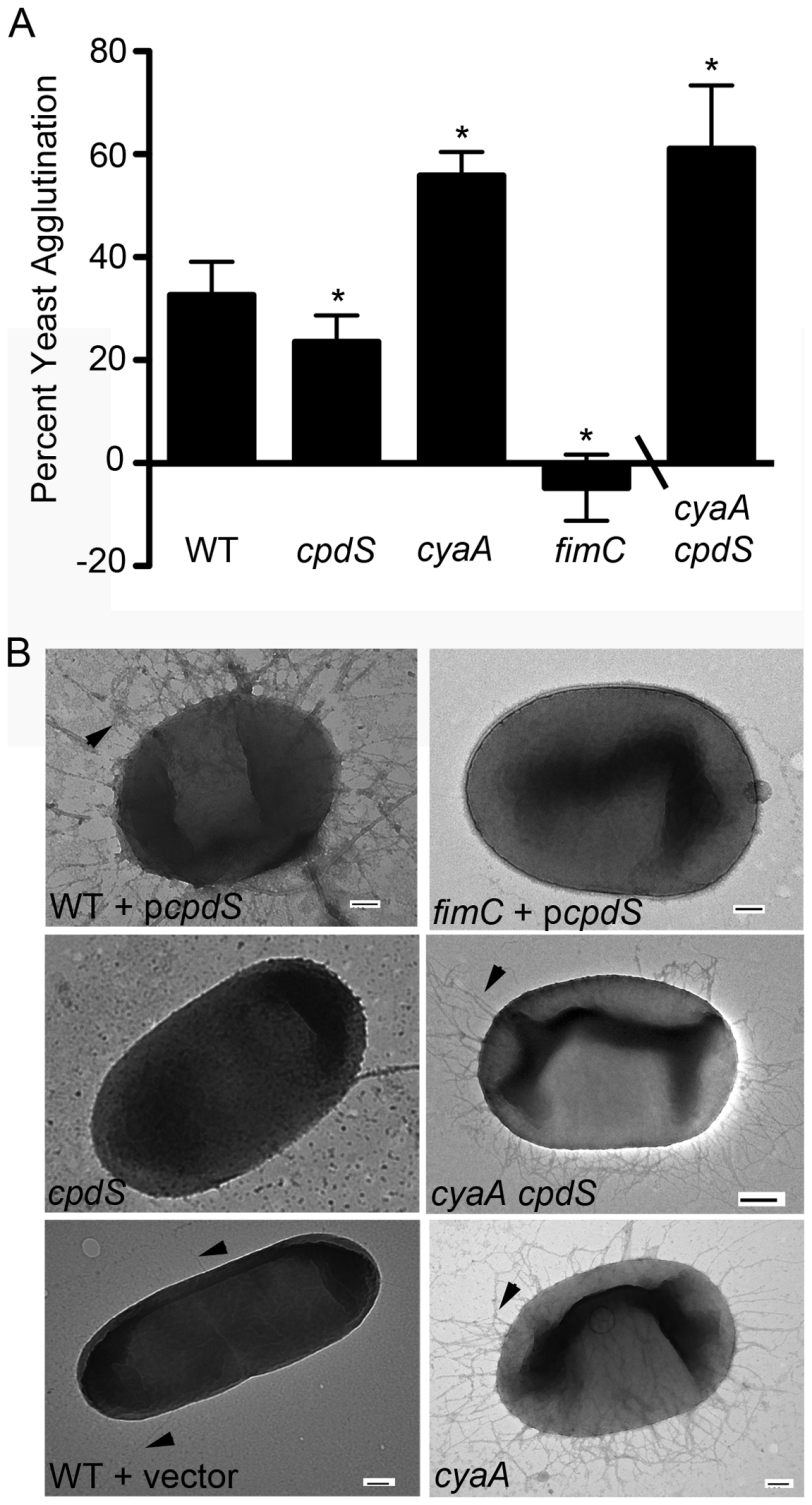
The *cpdS* mutant has reduced surface fimbriae. **A.** A yeast-agglutination assay was used to quantitate relative fimbriae expression. The asterisk indicates statistical difference from wild-type levels (n = 9, p<0.05, ANOVA with Tukey's post-test or Student's T-test). The *cyaA cpdS* double mutant data was from a different experiment (n = 6). **B.** Representative TEM micrographs of relevant strains. Black arrow-heads indicate fimbriae. Bar  = 100 µm.

A type I fimbriae yeast agglutination assay [34] was used to assess the defect of fimbriae production by the *cpdS* mutant. We observed a significant (p<0.05) decrease in the percent of yeast agglutination by the Δ*cpdS* mutant (23.6 ± 5.1%) compared to the WT (32.6 ± 4.7%) (Figure 5A). Whereas a negative control *fimC* mutant strain deficient for measurable fimbriae [35] produced no measurable agglutination, a positive control hyper-fimbriated *cyaA* mutant strain generated 55.9 ± 4.6% agglutination (Figure 5A).

A *cpdS cyaA* double mutant was isolated, see materials and methods, for epistasis analysis. The *cpdS cyaA* double mutant was highly proficient at agglutination of yeast (63.8 ± 5.6), similar to the *cyaA* mutant, and unlike the *cpdS* mutant. This result suggests that the *cyaA* mutation is epistatic to the *cpdS* mutation for fimbriae production.

Transmission electron microscopy (TEM) was used to directly observe the fimbriae on the surface of the WT and *cpdS* mutant (Figure 5B). Although it was difficult to differentiate between individual cells, on a population basis it was clear that the *cpdS* mutant produced fewer fimbria-like appendages (Figure 5B). Whereas 58.6% of WT cells exhibited at least one surface fimbria, only 32.1% of Δ*cpdS* cells had a clear fimbria (n≥80 cells per genotype, p<0.01 Fisher's Exact Test).

A dramatic hyper-fimbria phenotype was observed from the WT with multicopy expression of *cpdS* (p*cpdS*) similar to the *cyaA* mutant and *cpdS cyaA* double mutant (Figure 5B). Significantly more (p<0.01, Fisher's Exact Test) WT cells with the *cpdS* gene on a plasmid (88%, n = 479) had at least one surface fimbria compared to the WT cell with the vector alone (60.3%, n = 179). These structures were confirmed as fimbriae, and the hyper-fimbria phenotype conferred by *cpdS* on a plasmid was absent in a *fimC* mutant, as FimC is the usher protein required for generation of fimbriae (Figure 5B).

Yeast agglutination analysis confirmed that multicopy expression of *cpdS* induced fimbriae production (Figure S4). The WT strain with the vector alone exhibited significantly less yeast agglutination than the WT strain with pMQ171 (p*cpdS*). The WT strain with the same plasmid but with the N94A mutation (pMQ176) induced a similar amount of yeast agglutination as the WT with the empty vector supporting that cAMP-PDE activity was required for the increased surface fimbriae phenotype.

## Discussion

This study has demonstrated that *S. marcescens cpdS* (SMA3506) encodes a functional cAMP-PDE, although *S. marcescens* cAMP-PDE activity has been previously detected in the soluble fraction of crude cell extracts [48], [49]. Our results provided evidence that i) CpdS hydrolyzes cAMP to 5′-AMP (Figure 2A–B); ii) CpdS exhibits similar *in vitro* cAMP-PDE activity to CpdA homologs of *E. coli* and *P. aeruginosa* (Figure 2); iii) CpdS requires a conserved metal ion binding site through mutational analysis of *cpdS*-N94A (Figure 2–3); and iv) CpdS contains highly conserved amino acid residues and metal ion binding sites among class III cAMP-PDEs through multiple sequence alignment analysis (Figure S1). In addition, *in vivo* cAMP-PDE assays exhibited a 50-fold increase in intracellular cAMP levels in a *cpdS* deletion strain (Figure 3). We conclude that these genetic and biochemical data establish CpdS as a *bona fide* cAMP-PDE.

It is intriguing that isogenic *cpdA* mutant strains in *E. coli* [45] *S. typhimurium* [50], *V. vulnificus* [51], *Klebsiella pneumonia* [52], and *P. aeruginosa* [53] possess a wide range (∼2–30 fold increase) of intracellular cAMP compared to wild-type strains. This suggests that bacteria have adopted divergent cAMP-PDE-independent strategies for maintenance of cAMP concentrations. Indeed, there is compelling evidence that *E. coli* alternatively maintains intracellular cAMP through TolC-mediated cAMP export [54].

The biological relevance of bacterial cAMP-PDEs is inadequately understood, for which little functional significance has been identified except for cAMP homeostasis, sugar fermentation and transformation competence [45], [50], [53], [55]. Notably, CpdA homologs of *V. cholerae* [56] and *P. aeruginosa* are required for colonization efficiency and regulation of twitching-motility and virulence factors [53], [57], [58], respectively. The stringent control of cAMP levels is important in a wide variety of bacterial behaviors, thus we hypothesized that cAMP-PDEs could have an uncharacterized role in mediating other pathogenesis-associated phenotypes.

Through mutational, phenotypic, and microscopic analyses, we determined that CpdS is involved in the production of type I fimbrial adhesins and biofilm formation (Figure 4–5). These conclusions are supported by our findings that a *cpdS* deletion mutant is defective for biofilm formation but can be restored to wild-type levels with multicopy expression of *cpdS* (Figure 4). Furthermore, multicopy expression of *cpdS* in a wild-type background displays significantly elevated biofilm formation, however, biofilms were unaffected by over-expression of PDE-defective *cpdS*-N94A (Figure 4). We found that biofilm phenotypes were dependent on *fimC*, encoding a chaperone usher protein of the fimbrial operon *fimABCD*, similar to previous *S. marcescens* biofilm studies (Figure 5) [35]. Consistently, we observed a decrease in type I fimbriation for *cpdS* deletion mutants as determined through indirect (yeast agglutination) and direct (TEM) assays (Figure 5). Considering the importance of ACs and cAMP-CRP in type I fimbriae production and biofilm formation from prior work with *E. coli* and *S. marcescens* [24], [34], [35], it should be expected that cAMP levels are necessarily fine-tuned through cAMP-PDEs. It is plausible that the reciprocal interplay of ACs and cAMP-PDEs precisely modulates cAMP to permit flexibility in adjusting cAMP-regulated biofilm formation. To our knowledge, this report is the first description of a bacterial cAMP-PDE with the role of directing biofilm formation.

Ultimately, it will be important to identify the specific environmental signals, growth conditions, and/or regulatory proteins that modify cAMP-PDE effects on surface adhesins and biofilm formation. For instance, the potential influence of cAMP-CRP-dependent phase variation on type I fimbriation was not ascertained in this study as has been detailed in *E. coli* [23]. The orientation of the *fimA* promoter defines the phase as “on” or “off” which corresponds to the presence or absence of fimbrial transcription, respectively, with dramatic effects on early virulence and fimbriae-dependent bacterial colonization [59]. Although fimbrial phase-variation has not been described in *S. marcescens*, phase-related determinants such as the alarmone (p)ppGpp or sigma factor RpoS could feasibly have affected our observations of type I fimbriae [60], [61]. It is has been demonstrated through mutational analyses of *E. coli* catabolite repression machinery that cAMP-CRP is a negative regulator of *rpoS* expression [62], [63], and decreased *rpoS* expression has been shown in a *V. vulnificus cpdA* mutant [64]. Given the central role of RpoS in mediating the general stress response that contributes to antibiotic tolerance within biofilms [18], it is enticing to consider the possibly broader function of cAMP-dependent pathways in linking phase-dependent fimbriation with biofilm-mediated drug tolerance. Interestingly, it has been observed that cAMP-PDE mutants of *S. typhimurium* have increased antibiotic sensitivity, in which antibiotic and carbon sources were coupled through cAMP-dependent transporters [65], [66].

Bacterial signaling molecules and carbon source availability have a profound impact on the dispersal or detachment of biofilms [67]. A recent report by Huynh and colleagues highlighted the involvement of cAMP in glucose starvation-induced *P. aeruginosa* biofilm dispersal [68]. Because surface adhesins are largely involved in the primary attachment stage of surface colonization, we speculate that the observed importance of cAMP-PDEs on biofilm formation in our study was mediated at this early step. However, cAMP is emerging as a key signal in the later stages of biofilm development, namely biofilm dispersal, suggesting that cAMP flux may have a more important role in biofilm dynamics than formerly appreciated.

## Supporting Information

Figure S1 **Alignment of type III cAMP-Phosphodiesterase proteins.**
(DOCX)

Figure S2 **Purification and cAMP-PDE activity of recombinant CpdS.**
(TIFF)

Figure S3 **Live-Dead staining of WT and** ***cpdS*** **planktonic and biofilm cells.**
(TIF)

Figure S4 **Fimbriae-dependent yeast agglutination was induced by multicopy expression of** ***cpdS*** **.**
(TIFF)
