# Supplementary material for: Bacterial Cyclic AMP-Phosphodiesterase Activity Coordinates Biofilm Formation
Source: PLoS One. 2013 Jul 29;8(7):e71267. doi: 10.1371/journal.pone.0071267 (PMC3726613; doi:10.1371/journal.pone.0071267)
Supplement: Figure S1 — Alignment of type III cAMP-Phosphodiesterase proteins. (DOCX) [file pone.0071267.s001.docx]

E.c. MESLLTLPLAGEARVRILQIT**D**THLFAQKHEALLGVNTWESYQAVLEAIRPHQHEFDLIV 60

K.p. MESLLNLPLAGEARVRILQIT**D**THLFAEKHETLLGINTWDSYQAVLSAIHASQRPCDLIV 60

**S.m.** MESLFKLPVASGAAVRILQIT**D**THLFAGEHETLLGINTYRSYHAVLDAIQAQRRDVDLIV 60

H.i. MKNTFVYQAE-KPVIKLLQIT**D**PHLFKDESAELLGVNTQASFAQVLKEIQQENNEFDVIL 59

V.c. MK--VSSQSE-DSSIKLIQIT**D**THLFAAEDGSLLSVNTADSFAAVVAAIGEEQVEFDAIL 57

P.a. MSRHSNTPAT-DASVLLVQLS**D**SHLFAEDGARLLGMDTAHSLEKVVERVAREQPRIDLIL 59

M.t. -MHRLRAAEHPRPDYVLLHIS**D**THLIGGDRRLYGAVDADDRLGELLEQLNQSGLRPDAIV 59

. :::::*.**: . .::: :: : * *:

E.c. AT**GD**LAQDQSSAAYQHFAEGIASFRAPC----VWLP**GNHD**FQPAMYSALQDA--GISPAK 114

K.p. AT**GD**LAQDHSSAAYQHFAEGIASFAAPC----VWLP**GNHD**FQPAMYSTLQEA--GISPAK 114

**S.m.** AT**GD**LAQDHSQEAYRHFAAGIAQLPAPC----VWLP**GNHD**FQPAMVDALAAA--GIAPSK 114

H.i. AT**GD**LVQDSSDEGYIRFVEMMKPFNKPV----FWIP**GNHD**FQPKMAEFLNQP--PMNVAK 113

V.c. AT**GD**ISQDHTPESYQRFVRGIQPLKKAC----YWLP**GNHD**YKPSMHSVLPTQ--QIQAVE 111

P.a. AT**GD**VSQDGSLDSYTRFRRLSAPLDAPL----RWFA**GNHD**EREPMQRATE----GSDLLE 111

M.t. FT**GD**LADKGEPAAYRKLRGLVEPFAAQLGAELVWVM**GNHD**DRAELRKFLLDEAPSMAPLD 119

***: :. .* :: : *. **** : : .

E.c. RVFIGEQWQILLLDSQVFGVPHGELSEFQLEWLERKLADAPERHTLLLL**H**HHPLPAGCSW 174

K.p. RVFLGDRWQILLLDSQVFGVPHGELSDFQLEWLEHKLAEAPERYTLLLL**H**HHPLPAGCSW 174

**S.m.** QVLLGDNWQVLMLDSQVFGVPHGELSEYQLEWMERCLQAHPERYTLLLL**H**HHPLPSGCTW 174

H.i. HLLLGEHWQALLLDSQVYGVPHGQLSQHQLDLLKETLEKNPERYTLVVL**H**HHLLPTNSAW 173

V.c. HVLLGEHWQVVLLDSQVVGVPHGKLSEQQLQLLDNKLTEYPERHTLVLL**H**HHPLLVGSAW 171

P.a. QVVDVGNWRVVLLDSSIPGAVPGYLEEDQLELLRRAIDSAGERFLLVSF**H**HHPVPIGSDW 171

M.t. RVCMIDGLRIIVLDTSVPGHHHGEIRASQLGWLAEELATPAPDGTILAL**H**HPPIPSVLDM 179

:: : ::**:.: * * : ** : . : :: :** :

E.c. LDQHSLRNAGELDTVLAKFPHVKYLLC**GHIH**QELDLDWNGRRLLATPSTCVQFKPHCSN- 233

K.p. LDQHSLRNAGALDSALSAWPRVKHLLC**GHIH**QELDLDWNGRRMMATPSTCVQFKPHCAN- 233

**S.m.** LDQHSLRNPHMLGAILLRYPKVNTVVC**GHIH**QDLDLEWQGRRLLATPSTCVQFKPHCTN- 233

H.i. LDQHNLRNSHELAEVLSPFTNVKAILY**GHIH**QEVNSEWNGYQVMATPATCIQFKPDCQY- 232

V.c. LDQHTLKESERFWDVVAKHSNVKAILC**GHVH**QDMDRIHLGARVMATPSTCVQFKPNSQD- 230

P.a. MDPIGLRNPQALFDLLAPYPQVRCLLW**GHIH**QEFDRQRGPLRLLASPSTCVQFAPGSSD- 230

M.t. AVTVELRDQAALGRVLRG-TDVRAILA**GHLH**YSTNATFVGIPVSVASATCYTQDLTVAAG 238

*:: : : . *. :: **:* . : : .:.:**

E.c. -FTLDTIAPGWRTLELHADGTLTTEVHR---------LADTRFQPDTASEGY-------- 275

K.p. -FTLDTVSPGWRWLELHPDGTLTTEVCR---------LEGAAFHPDIASEGY-------- 275

**S.m.** -FTIDDVSPGWRYLDLLPDGRVETQVFR---------LENDDFRPDMDSDGY-------- 275

H.i. -FSLDTLQPGWREIELHSDGSIRTQVKR---------IQQAEFLPNMQEEGY-------- 274

V.c. -FALDNCSPGWRELTLHADGQVSTQVKR---------LKQGRFLPDFNSNGY-------- 272

P.a. -FTLDRLAPGYRWLRLHDDGRLETGISR---------VDDVVFEVDYDTAGY-------- 272

M.t. GTRGRDGAQGCNLVHVYPDTVVHSVIPLGGGETVGTFVSPGQARRKIAESGIFIEPSRRD 298

* . : : * : : : : . *

E.c. --------------------

K.p. --------------------

**S.m.** --------------------

H.i. --------------------

V.c. --------------------

P.a. --------------------

M.t. SLFKHPPMVLTSSAPRSPVD 318

**Figure S1. Alignment of type III cAMP-Phosphodiesterase proteins.** Protein sequences were aligned using CLUSTAL 2.1 multiple sequence alignment. E.c. = *Escherichia coli*; K.p. = *Klebsiella pneumoniae*; S.m. = *Serratia marcescens*; H.i. = *Haemophilus influenzae*; V.c. = *Vibrio cholerae*; P.a. *= Pseudomonas aeruginosa*; M.t. = *Mycobacterium tuberculosis*. The (D-(X)n-GD-(X)n-GNH[E/D]-(X)n-H-(X)n-GHXH) purple phosphatase family active site residues are in bold and the conserved asparagine residue involved in metal binding (N94) is underlined.
