# Supplementary figures and images for: Bacterial Cyclic AMP-Phosphodiesterase Activity Coordinates Biofilm Formation
Source: PLoS One. 2013 Jul 29;8(7):e71267. doi: 10.1371/journal.pone.0071267 (PMC3726613; doi:10.1371/journal.pone.0071267)

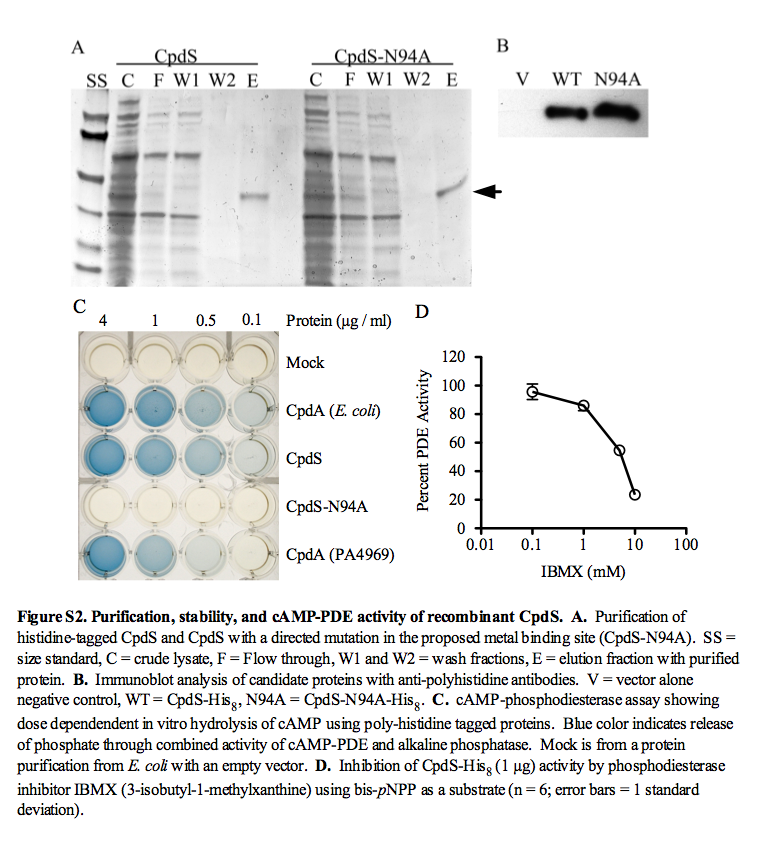

Supplement: Figure S2 — Purification and cAMP-PDE activity of recombinant CpdS. (TIFF) [file pone.0071267.s002.tif]

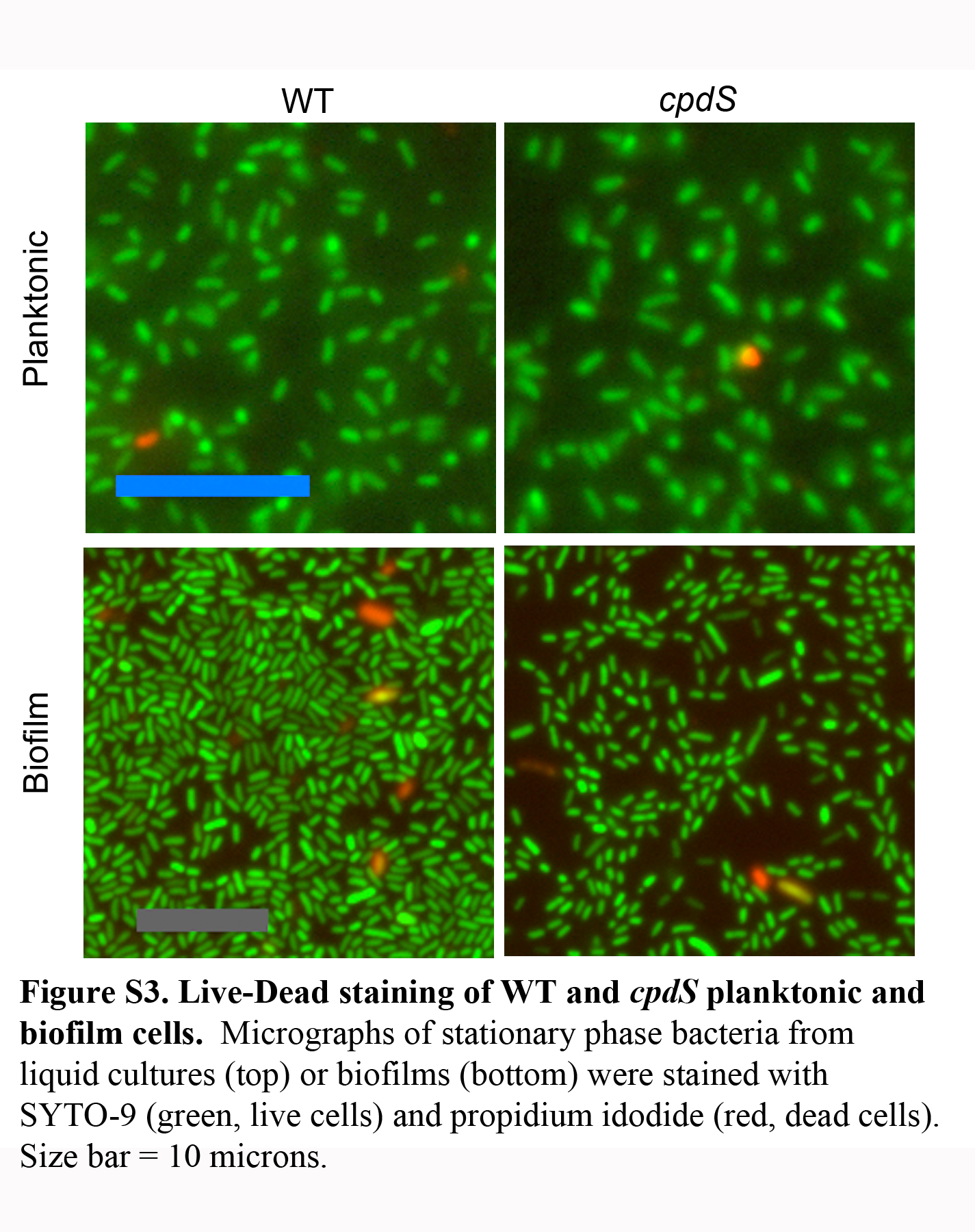

Supplement: Figure S3 — Live-Dead staining of WT and cpdS planktonic and biofilm cells. (TIF) [file pone.0071267.s003.tif]

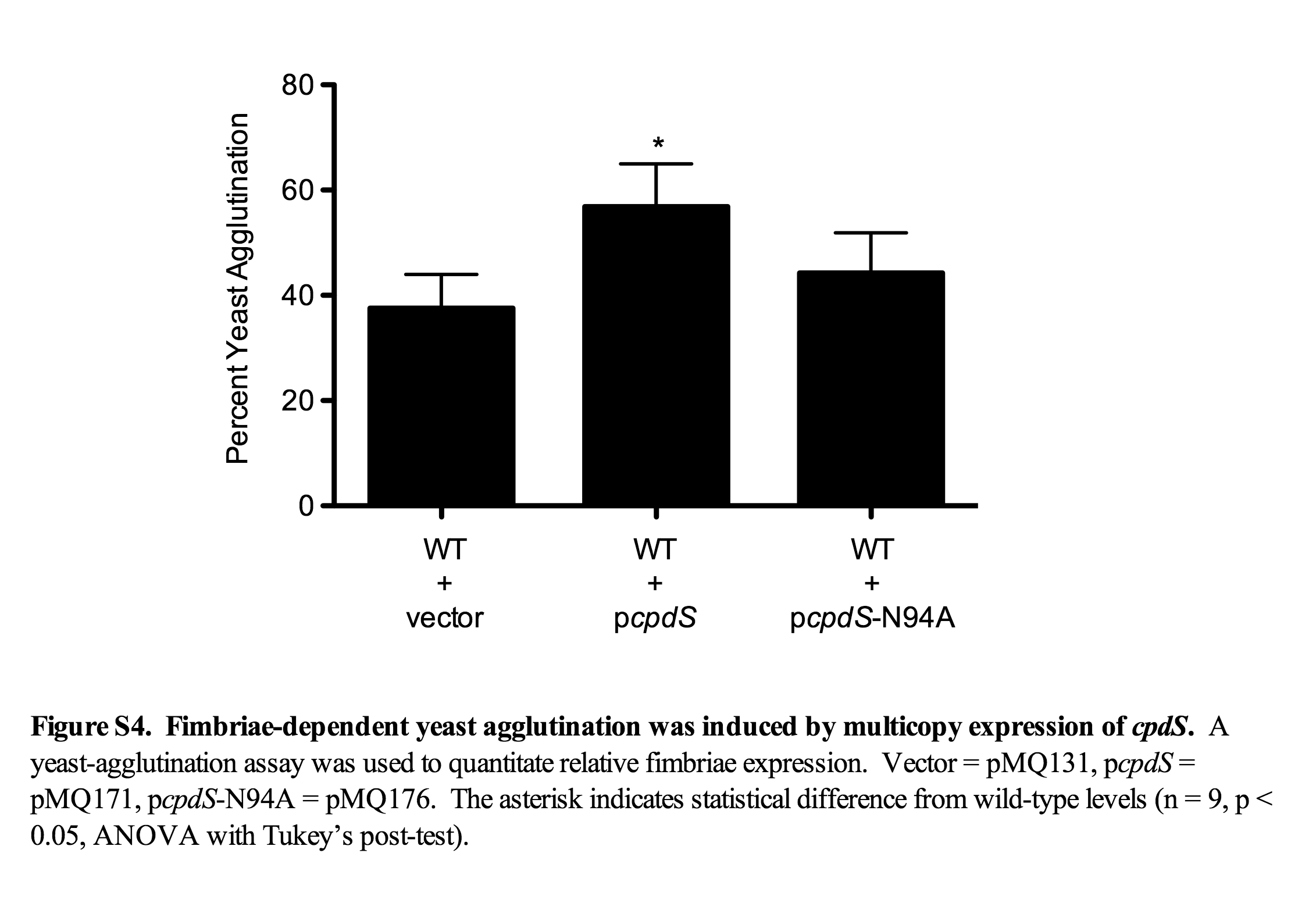

Supplement: Figure S4 — Fimbriae-dependent yeast agglutination was induced by multicopy expression of cpdS . (TIFF) [file pone.0071267.s004.tif]
